# First clinical isolate of *Escherichia coli* harboring *mcr*-1 gene in Mexico

**DOI:** 10.1371/journal.pone.0214648

**Published:** 2019-04-04

**Authors:** Jocelin Merida-Vieyra, Agustín De Colsa- Ranero, Patricia Arzate-Barbosa, Eduardo Arias- de la Garza, Alfonso Méndez-Tenorio, Jazmin Murcia-Garzón, Alejandra Aquino-Andrade

**Affiliations:** 1 Molecular Microbiology Laboratory, Instituto Nacional de Pediatria (National Institute of Pediatrics INP), Mexico City, Mexico; 2 Pediatric Infectious Diseases Department, INP, Mexico City, Mexico; 3 Clinical Bacteriology Laboratory, INP, Mexico City, Mexico; 4 Healthcare Associated Infection Advisory Committee, INP, Mexico City, Mexico; 5 Escuela Nacional de Ciencias Biologicas, Instituto Politecnico Nacional (National School of Biological Sciences, National Polytechnic Institute), Mexico City, Mexico; Universidade Nova de Lisboa, PORTUGAL

## Abstract

Our aim in this report was to describe the characteristics of the first clinical isolate of *Escherichia coli* (EC-PAG-733) harboring the *mcr-*1 gene found in Mexico. This isolate was obtained from a fecal sample from a young child with an oncological condition. We obtained the whole-genome sequence using next-generation sequencing and analyzed the sequence by bioinformatics tools. EC-PAG-733 was resistant to third- and fourth-generation cephalosporins and was susceptible to all carbapenems and amikacin; it was also resistant to ciprofloxacin, levofloxacin, gentamicin and colistin at a minimum inhibitory concentration (MIC) of 4 μg/mL. This isolate was classified as O11:H25-ST457. EC-PAG-733 harbored an ESBL type CTX-M-55 as well as several virulence factors that have been associated with Enteroaggregative *Escherichia coli* (EAEC). The *mcr-*1 gene was located within an IncI*2* plasmid. The results of this whole genome shotgun project were deposited in DDBJ/ENA/GenBank under the accession number QKXE00000000.

## Introduction

Since its discovery, the resistance to polymyxins that is mediated by the *mcr-*1 gene has been considered to be an emerging resistance mechanism of global concern [1]. One of the main reasons for this alarm is that polymyxins are one of the last treatment options for infections caused by carbapenem-resistant enterobacteria, and the underlying mechanism of this resistance is transmitted by plasmids [2,3].

The use of colistin for growth promotion and for prophylaxis in farm animals has especially contributed to the emergence of colistin resistance; furthermore, its use for the treatment of infections due to carbapenem-resistant bacilli, such as *Pseudomonas aeruginosa*, *Acinetobacter baumannii* and Enterobacteriaceae, has been increasing [2]. In Latin America, the rate of colistin resistance resulting from this group of bacteria is estimated to be 1.5% [4]. In Mexico, the frequency of resistance to colistin in Gram negative bacilli is unknown.

*Escherichia coli* strains containing the *mcr-*1 gene have been isolated from animal food products, humans and other animal sources around the world, including in many Latin America countries [5–8]. There are several genetic variants of the *mcr* gene that could be carried on plasmids or integrated into chromosomes that are mainly distributed within members of *Enterobacteriaceae* [6]. In Mexico, only one study has reported the identification of an *E coli* isolate containing the *mcr*-1 gene, which was obtained from a pig stool sample [9].

In 2017, we established a surveillance program in our hospital to detect emerging resistance mechanisms in Gram negative bacilli, which allowed us to identify an isolate of *E*. *coli* containing the *mcr*-1 gene in a pediatric patient. The aim of this report was to characterize a clinical isolate of *E*. *coli* (EC-PAG-733) that contains the *mcr-*1 gene and to describe the epidemiological features of the patient from whom this strain was obtained.

## Materials and methods

### Ethics statement

This study was approved by the biosecurity, ethics, and research committees of the INP (IRB:00008064 and IRB:00008065) Number 2018/017. The Review Board exempted this study from the need to request informed consent because the isolate described in the study was obtained by routine procedures and did not affect the patient. There is no identifying information in this document or in any supporting information file that could compromise patient confidentiality or participant privacy.

### Microbiological procedures

An isolate of *E*. *coli* known as EC-PAG-733 was obtained from a fecal sample from a pediatric patient in 2017. We obtained the identification, and susceptibility profile using the Phoenix Automated Microbiology System (Becton Dickinson, USA). We determined the MIC of colistin by broth microdilution method. The results were interpreted according to the guidelines of the Clinical and Laboratory Standards Institute (CLSI). The detection of extended-spectrum beta-lactamases (ESBL) was performed using a double-disk diffusion test [10].

### Molecular procedures

We obtained the total DNA using a QIAamp DNA Mini kit (QIAGEN, Hilden, Germany) according to the manufacturer’s instructions. As part of the general procedures used for our surveillance program, we first amplified *bla*_CTX-M-1,_ *bla*_CTX-M-2,_ *bla*_CTX-M-9,_ *bla*_SHV,_ *bla*_TEM,_ *bla*_LAT,_ and *bla*_DHA_ genes by PCR [11] with a GenAmp PCR System 9700 (Applied Biosystems, Foster City, CA, USA) and AmpliTaq Gold 360 MasterMix (Applied Biosystems). In addition, we searched several fluoroquinolone resistance genes, such as *aac(6´)-Ib-cr*, *qnr*A, *qnr*B and *qnr*S [12,13]. We amplified the *mcr*-1 gene using previously reported primers [1]. The obtained fragment was sequenced using an ABI Prism 310 analyzer. The sequence was analyzed with the blastn algorithm [14,15]. To obtain the phylogroup of EC-PAG-733, we used the method described by Clermont *et al*. [16]. Multilocus sequence typing (MLST) was performed by the amplification of seven housekeeping genes [17]. The sequences were analyzed using the program and database available at http://enterobase.warwick.ac.uk/species/ecoli/allele_st_search [18].

Subsequently, we obtained the whole genome sequence with the Ion Torrent S5 XL sequencer (Thermo Fisher Scientific, Waltham, MA, USA). The genome was assembled and annotated with the Rapid Annotation by Subsystem Technology (RAST) server and analyzed using bioinformatics tools to identify any virulence genes, acquired antimicrobial resistance genes, the serotype; to type the plasmids, to confirm the MLST results and to determinate the probability to be a human pathogen [19–25]. We analyzed the plasmid that contained the *mcr*-1 gene with blastn and MAUVE 2.4.0 [14, 15, 26].

## Results

The EC-PAG-733 isolate was obtained from a pediatric patient diagnosed with pre-B acute lymphoblastic leukemia (L1). The patient had been admitted several times due to febrile neutropenia that required antimicrobial treatments prior to the isolation of EC-PAG-733. The patient required HSCT to treat her hematological disorder.

The first admission for HSCT required a total of 32 days of hospital stay; the patient received an umbilical cord transplant, which resulted in the loss of the graft; the patient had septic shock that was treated with cefepime for 14 days. During the second admission (35 days), the child received a new allogeneic transplant from a nonrelated donor; the graft was successful 10 days after the transplant. The patient presented with febrile neutropenia and was treated with meropenem for 14 days.

During the third admission (60 days), the patient presented with febrile neutropenia, a systemic adenoviral infection, diarrhea that was caused by *Clostridium difficile* and lymphoproliferative disease due to Epstein Barr virus. During this hospitalization, the patient received antimicrobial treatments with ceftriaxone (10 days), cefepime (3 days), and meropenem (5 days), but not polymyxins.

On day 34 after the third admission, the child presented with noninvasive diarrhea, which was self-limiting after 7 days. No pathogen was identified by a molecular gastrointestinal panel (xTAG Gastrointestinal Pathogen Panel, Luminex Molecular Diagnostics, Toronto, Canada). A stool culture was performed, and the unique isolate was identified as an *E*. *coli strain* (EC-PAG-733).

The EC-PAG-733 isolate was susceptible to all carbapenems and amikacin, and it was resistant to third and fourth generation cephalosporins (the phenotypic ESBL test was positive), fluoroquinolones, and gentamicin; the MIC of colistin was 4 μg/mL, so this isolate was classified as a non-wild-type strain (Table 1).

**Table 1:** Susceptibility profile of EC-PAG-733.

Agent	AMP	SAM	CFZ	FOX	CAZ	FEP	PTZ	MEM	IPM	ETP	AMK	CIP	LEV	GEN	SXT	CL
MIC*	>16	>16/8	>8	≤8	>16	>16	>64/4	<1	<1	<1	≤8	>2	≥4	≥8	>2/38	4

*In μg/mL. AMP: ampicillin; SAM: ampicillin-sulbactam; CFZ: cefazoline; FOX: cefoxitin; CAZ: ceftazidime; FEP: cefepime; PTZ: piperacillin/tazobactam; MEM: meropenem; IPM: imipenem; ETP: ertapenem; CIP: ciprofloxacin; LEV: levofloxacin; AMK: amikacin; GEN: gentamicin; SXT: trimethoprim-sulfamethoxazole; CL: colistin.

Of all the genes that we searched by PCR, we only detected the *bla*_CTX-M-1_ group and *mcr*-1. Based on the MLST results, we concluded that EC-PAG-733 was a ST457 clone, which was confirmed by the analysis of the complete genome. The isolate was classified as a member of the F phylogroup, and its serotype was O11:H25 and contained *fim*H145. Furthermore, the isolate was predicted as a human pathogen (probability: 0.931; number of matches with pathogenic families: 533).

EC-PAG-733 (S1 Supporting Information) contained several genes that conferred resistance to aminoglycosides (*aac(3)-IIa* and *aadA1*), beta-lactams (*bla*_CTX-M-55_), chloramphenicol (*floR*), sulfonamides (*sul3*), trimethoprim (*dfrA14*), tetracyclines (*tetA*), and colistin (*mcr*-1). Furthermore, we identified mutations in *gyr*A (S83L, D87) and *par*C (S80I) that are associated with resistance to fluoroquinolones.

**Fig 1:**
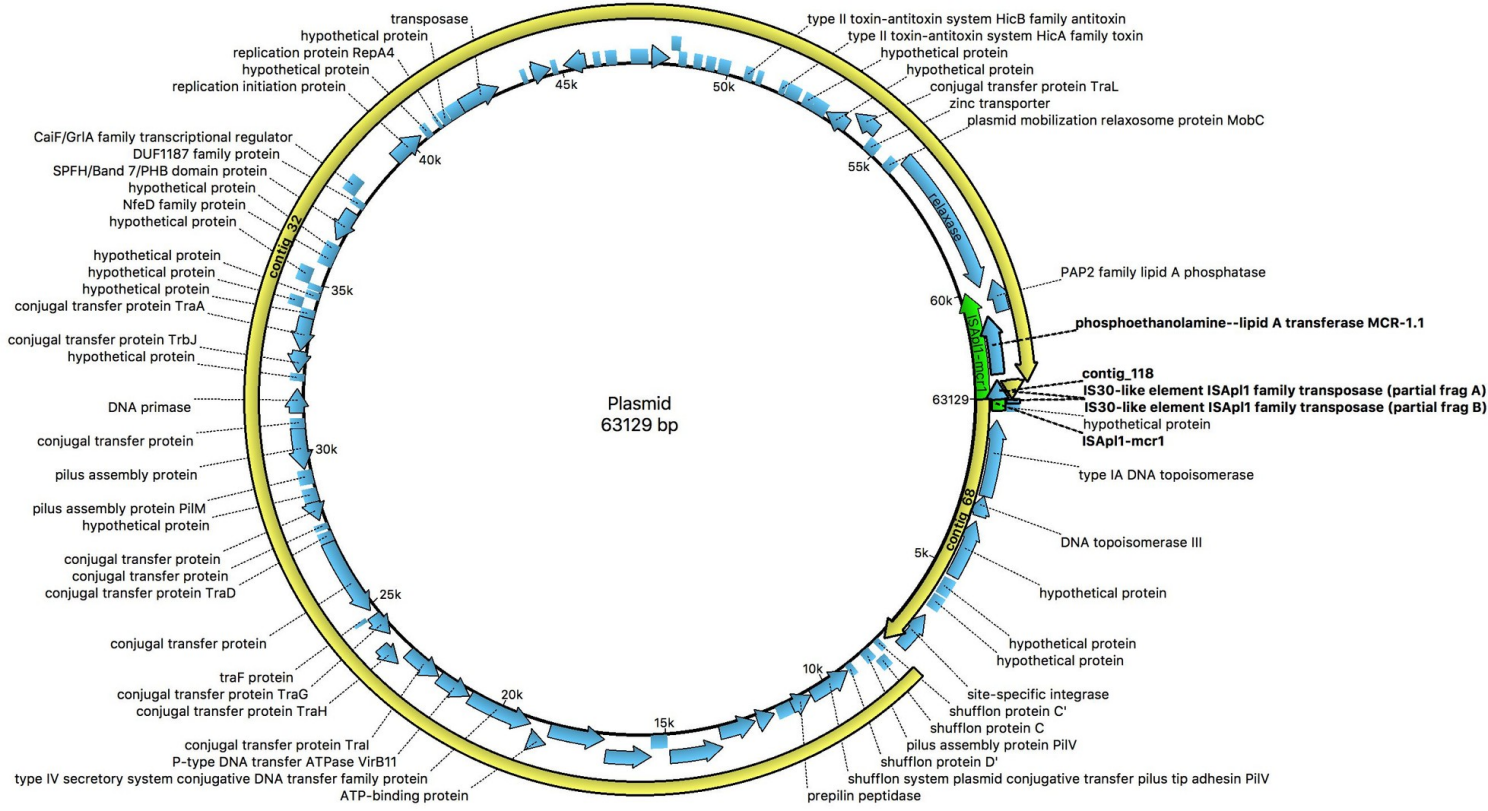
The circular map of the pEC-PAG-733 genome (Ugene). The location of the *mcr-*1 gene is shown.

**Fig 2:**
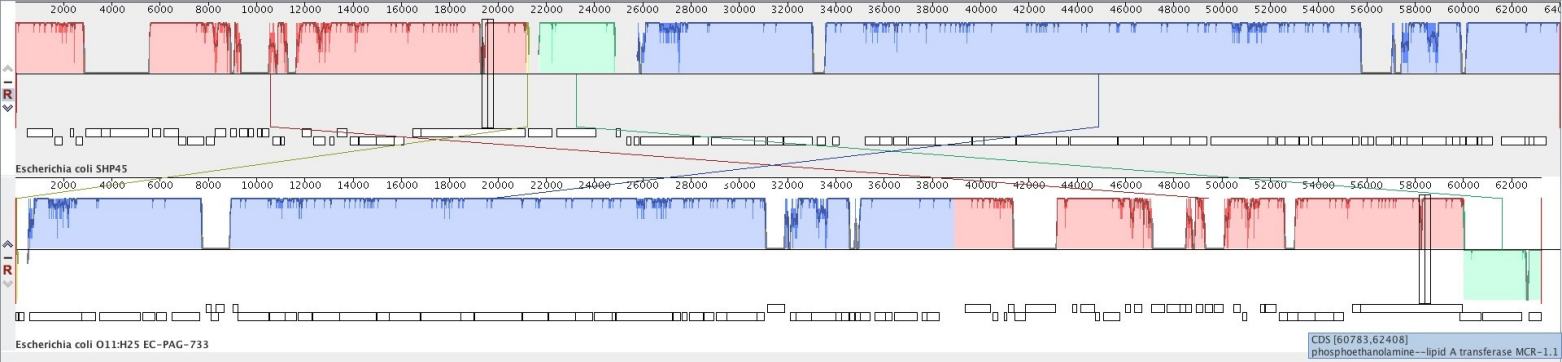
Alignment of pEC-PAG-733 and pHNSHP45 (accession KP347127.1) produced using MAUVE software.

Using plasmid finder software, we identified the *mcr*-1 gene, which was encoded in a plasmid that belongs to IncI2 within the EC-PAG-733 genome. Within the sequence that was submitted to and annotated by the NCBI, we observed that three contigs, 118, 32 and 68, that comprised practically the entire plasmid (S2 Supporting Information). The plasmid was 63,129 bp in length; the IS*Apl1* insertion sequence was located upstream of the *mcr*-1 gene (Fig 1 and S3 Supporting Information). We found that the plasmid exhibited high identity with other plasmids that contain the *mcr*-1 gene, including pMCR-H9, p14EC033a, plasmid A, and pHNSHP45, based on plasmid alignment (S4 Supporting Information). We compared the pEC-PAG-733 genome with the sequence of the first plasmid that was reported to harbor the *mcr-1* gene (Fig 2).

The isolate harbored the virulence-related genes *eilA* (HilA family transcriptional regulator), *iss* (increased serum survival), *air* (Enteroaggregative immunoglobulin repeat protein), *cma* (colicin M) and *lpfA* (long polar fimbriae).

We deposited the sequence that was obtained from the whole genome shotgun project in DDBJ/ENA/GenBank under the accession number QKXE00000000 (EC-PAG-733; Supporting Information).

## Discussion

There is a paucity of data regarding the presence of *E*. *coli* containing the *mcr*-1 gene in pediatric patients, either in carriers or those with clinical infections [27,28]. We described the epidemiological characteristics of the patient from whom EC-PAG-733 was isolated. The presence of *E*. *coli* containing *mcr*-1 both in infected patients and in healthy carriers has been associated with several risk factors, including male gender, the presence of immunosuppression, previous hospital stays and the use of antibiotics, especially carbapenems and fluoroquinolones [29, 30]. The patient was subject to multiple well-known risk factors, such as previous carbapenem treatment and the presence of underlying diseases that resulted in immunosuppression (leukemia and HSCT). The patient did not receive colistin treatment during any of the hospital stays; however, strains of *E*. *coli* containing *mcr*-1 that were isolated from immunosuppressed patients who did not receive colistin treatment have also been reported [8].

One very relevant epidemiological factor was the fact that this patient had close contact with relatives dedicated to raising pigs, which could be the source of this pathogen; living near a farm has been found to be a risk factor that increases the probability of colonization for healthy individuals [30].

We observed a clear correlation between the resistance phenotype and genotype. We did not find carbapenem resistance. The CTX-M-55 beta-lactamase confers resistance to all beta-lactams except carbapenems, which explains the resistance of EC-PAG-733 to cefazoline, cefoxitin, ceftazidime, and cefepime. The gentamicin resistance could be explained by the presence of the *aac(3)-IIa* and *aadA1* genes. In addition, in the genome of EC-PAG-733, we found mutations in the *gyr*A and *par*C genes that have been closely associated with resistance to fluoroquinolones [31], which explained the MICs of ciprofloxacin and levofloxacin of ≥2 and ≥4 μg/mL, respectively.

EC-PAG-733 belongs to the F phylogroup, which is closely related to the B2 phylogroup; some clonal complexes (CC) derived from the F phylogroup are considered emerging lineages that are associated with resistance and *mcr*-1, such as the epidemic strains of the ST648 complex [32,33]. This finding indicates that EC-PAG-733 could be considered as a pathogen; in addition, other clones of *E*. *coli*, such as ST457, could also contribute to the dissemination of the *mcr*-1 gene.

Several clones have been associated with the spread of the *mcr*-1 gene, which suggests that horizontal transmission is the mechanism that has allowed for its worldwide dissemination [34]. In Latin American countries, clinical isolates of *E*. *coli* with the *mcr*-1 gene have been reported in many countries, including Brazil [8,35], Venezuela [36], Argentina [37], and Ecuador [7]; these isolates belong to several different STs, which indicates that the dissemination of the *mcr-*1 gene has been the result of several clonal origins in this region of the world. None of these studies reported the presence of the ST457 clone. In Mexico, there was only one previous report of *E*. *coli* containing the *mcr*-1 gene, which was an *E*. *coli* ST44 strain that was isolated from a stool sample from a pig. This isolate and EC-PAG-733 shared several resistance genes, including CTX-M-55, *aac(3)-IIa*, *sul*3, *tetA*, *dfrA14*, and mutations in *gyr*A and *par*C [9].

*E*. *coli* ST457 containing the *mcr*-1 gene has been isolated from carriers, patients and animals in other parts of the world. Some of these reports have indicated that the *mcr-1* gene coexists with other acquired resistance mechanisms, as was found in EC-PAG-733, which harbored both *mcr-1* and CTX-M-55. In Taiwan, in 2014, an *E*. *coli* ST457 isolate from a blood culture was reported that harbored the CTX-M-1 group ESBL and *mcr*-1 genes [38]. Other groups and families of beta-lactamases have been found in *E*. *coli* ST457 with the *mcr*-1 gene, as was described for an isolate obtained from a bile culture with carbapenem resistance, but no carbapenemase production, and also carrying CTX-M-14 (CTX-M-9 group), CMY-2 and TEM-1 [39].

Other study has documented four isolates of ST457 *E*. *coli*, which were found in cattle with mastitis, that harbored CTX-M-27; these isolates had some molecular characteristics in common with EC-PAG-733, such as the presence of *aac(3)-II* and *dfrA*14, which are related to aminoglycoside and trimethoprim resistance, respectively [40]; in addition, ST457 clone strains without resistance genes have been isolated from healthy Korean carriers [41].

*E*. *coli* isolates containing the *mcr*-1 and carbapenemase genes have been described mainly with enzymes NDM, KPC and OXA-48 families [42]. EC-PAG-733 was not carbapenem resistant. In *E*. *coli* ST457, in particular, the coexistence of *mcr*-1 with these enzymes has not been reported. However, the appearance of a *mcr*-1 gene-containing isolate in our hospital resulted in an epidemiological alert because we had recently reported six cases of infection produced by carbapenemase-producing enterobacteria [43]. The horizontal transfer of the *mcr*-1 gene into carbapenem-resistant *Enterobacteriaceae* would severely limit antimicrobial treatment options, so it is important to strengthen our surveillance of bacteria exhibiting this type of resistance mechanism. In addition, the *mcr*-1 gene has also been associated with the high-risk clone ST131. *E*. coli ST131 strains containing *mcr*-1 have been isolated from several types of samples, including urine, blood, abscesses and peritoneal fluid [8,38,41,44]. In our hospital, > 40% of ESBL-producing *E*. *coli* isolates were ST131 (unpublished data).

We located the *mcr-*1 gene into an IncI2 plasmid; it belonged to the same incompatibility group of pHNSHP45 as described by Liu *et al*. in the first report describing *mcr*-1 in China [1]. There is evidence that as many as 34% of the plasmids that possess the *mcr*-1 gene belong to IncI2 group. This group has been described frequently in Asia; moreover, IncI2 plasmids containing *mcr*-1 have also been found in other countries, including the USA [3, 44,45]. Furthermore, within a single animal isolate that was obtained in Mexico, the *mcr-1* gene was located in an Incp0111 plasmid [9]. These findings indicate that could be several clones and plasmids that participate in the dissemination of the *mcr*-1 gene.

EC-PAG-733 belongs to the O11 serogroup, which has been associated with diarrheagenic *E*. *coli*, extraintestinal pathogenic *E*. *coli* (ExPEC), and avian pathogenic *E*. *coli* (APEC) [46,47]. It also contains several virulence factors and belongs to the F phylogroup. We could not establish that the diarrhea of the patient was caused by EC-PAG-733, since it did not correspond to a classic diarrheagenic *E*. *coli* type; however, this strain was the unique isolate identified from the stool culture, and we did not detect any other pathogens in the gastrointestinal panel. Moreover, bioinformatic analysis indicated that this isolate had characteristics that would allow it to be a human pathogen.

The virulence factors in clinical isolates containing *mcr*-1 have not been widely examined [48,49]. Therefore, it was of great interest to us to describe the virulence genes in EC-PAG-733. The isolate was found to contain the *eilA* gene, which encodes a homolog of the transcriptional regulator HilA and may contribute to the virulence of EAC [50]. This regulator has also been found in EAEC isolates from carriers and patients with diarrhea; the frequency of this gene in these groups was not significant [51].

EC-PAG-733 was also found to harbor the *iss* gene, which is involved in resistance to the avian complement system [52]. This gene was found in 47% of *E*. *coli* isolates that produced CTX-M-1 [11]. In ExPEC isolates obtained from blood cultures, this gene was detected at a lower frequency (9.3%) [53]; within a collection of 85 uropathogenic *E*. *coli* isolates that were obtained from patients with urinary tract infections, the *iss* gene was detected at a 20% frequency; these isolates were classified as belonging to either the A, B2 or D phylogroups [54]. Additionally, the *iss* gene has been found in Shiga toxin-producing *E*. *coli* that were isolated from patients with and without bloody diarrhea and was more frequently found in isolates that did not produce bloody diarrhea; these results were statistically significant [55]. This gene has also been associated with APEC [46].

In EAEC, the *air* gene is regulated by HilA. This gene was identified at a frequency of 39.5% in isolates from this pathogroup. In HEp-2 cells, Air was shown to contribute to adherence [50], but did not participate in colonization within a mouse model [56]; however, when the frequency of this gene in EAEC isolates obtained from carriers and patients with diarrhea was compared, a significant difference was observed [51].

The *cma* gene, which encodes colicin M, was found in EC-PAG-733. This virulence factor was also detected in an *E*. *coli* ST3204 strain that coproduced NDM-16 and MCR-1 [49]. Colicin M, which is a toxin that is secreted by the bacteria to kill other bacteria of the same species or related species [57], was detected in EC-PAG-733.

In addition, EC-PAG-733 was found to harbor the *lpfA* gene, which encodes a fimbrial protein that is associated with the invasion of epithelial tissue. This gene has been found in several clones of *E*. *coli* as well as Shiga toxin-producing *E*. *coli* isolated from children. Additionally, the *lpfA* gene was found more frequently in samples of nonbloody diarrhea than in samples of bloody diarrhea [55]. The *lpfA* gene has also been described as a virulence factor within a hybrid enteroagreggative *E*. *coli* O104:H4 that had acquired the Shiga-toxin 2 gene [58].We proposed that the coexistence of virulence factors, such as *eilA*, *iss*, *air*, *cma* and *lpfA*, in EC-PAG-733 could enhance its role as a pathogen.

## Conclusions

To the best of our knowledge, this is the first study that has reported the characteristics of a clinical isolate of *E*. *coli* harboring the *mcr*-1 gene that was obtained from a Mexican pediatric patient. The patient had multiple risk factors for being a carrier or suffering from infectious disease caused by multidrug-resistant microorganisms, such as a prolonged hospital stay, previous treatment with antibiotics and immunosuppression.

The frequency of community-acquired infections caused by bacteria harboring *mcr*-1 in our country is almost completely unknown because first and second level care hospitals do not have the diagnostic tools required to accurately determine resistance to polymyxins or the genetic characteristics of isolates.

In our hospital, we consider it is essential to increase the surveillance of emerging mechanisms of resistance, such as *mcr*-1, in a way that involves all healthcare professionals who participate in the diagnosis, treatment, control and prevention of infectious diseases.

## Supporting information

S1 Supporting Information EC-PAG-733 genome. (RAR)

S2 Supporting Information pEC-PAG-733. (FAS)

S3 Supporting Information EC-PAG-733 and IS*ApI1* alignment. (TXT)

S4 Supporting Information Plasmids alignment. (RAR)
